# Supplementary material for: Exploiting Dual-Gate Ambipolar CNFETs for Scalable Machine Learning Classification
Source: Sci Rep. 2020 Mar 31;10:5735. doi: 10.1038/s41598-020-62718-0 (PMC7109128; doi:10.1038/s41598-020-62718-0)
Supplement: Supplementary file 1 — Supplementary Information. [file 41598_2020_62718_MOESM1_ESM.pdf]

Supplementary Information

Exploiting Dual-Gate Ambipolar CNFETs for Scalable Machine Learning Classification

Farid Kenarangi[1,*], Xuan Hu[2], Yihan Liu[3], Jean Anne C. Incorvia[3], Joseph S. Friedman[2], and Inna Partin-Vaisband[1]

[1]University of Illinois at Chicago, Chicago, 60607, USA
[2]University of Texas at Dallas, Richardson, 75080, USA
[3]University of Texas at Austin, Austin, 78712, USA
[*]fkenar2@uic.edu

Code and Simulation Instructions

All the simulations in this paper are performed using Cadence (R) Virtuoso (R) Spectre (R) Circuit Simulator. To help readers to reproduce results, step by step instructions along with all the code and simulation files are uploaded to an online public GitHub repository. The repository can be accessed at https://github.com/faridken/AP-CNFET-based-ML. The repository comprises of six directories as listed below:

1. data: containing the data corresponding to the input images for testing the classifier during inference.
2. images: containing the screenshots of the simulation environment and results.
3. lib: containing the library of AP-CNFET device used within the manuscript.
4. log: containing the log file generated by Cadence for classification of 10,000 MNIST test images.
5. output: containing the SPICE simulation results and script for extracting the accuracy.
6. src: containing the main NETLIST file of the circuit proposed for classifying the MNIST dataset.
